# Modulation of Anthocyanin Biosynthesis-Related Genes during the Ripening of *Olea europaea* L. cvs Carolea and Tondina Drupes in Relation to Environmental Factors

**DOI:** 10.3390/ijms24108770

**Published:** 2023-05-15

**Authors:** Michele Ferrari, Antonella Muto, Leonardo Bruno, Innocenzo Muzzalupo, Adriana Chiappetta

**Affiliations:** 1 Department of Biology, Ecology, and Earth Sciences, University of Calabria, 87036 Arcavacata di Rende, Italy; michele.ferrari@unical.it (M.F.); antonella.muto@unical.it (A.M.); leonardo.bruno@unical.it (L.B.); 2 CREA—Council for Agricultural Research and Agricultural Economy Analysis, Forestry and Wood Research Center, 87036 Rende, Italy; innocenzo.muzzalupo@crea.gov.it

**Keywords:** environmental temperature responses, fruit ripening, MYB transcription factor, *Olea europaea*, olive fruit

## Abstract

Anthocyanins protect plants against various biotic and abiotic stresses, and anthocyanin-rich foods exert benefits on human health due to their antioxidant activity. Nevertheless, little information is available on the influence of genetic and environmental factors on the anthocyanin content in olive fruits. Based on this consideration, the total anthocyanin content, the genes involved in anthocyanin biosynthesis, and three putative R2R3-MYB transcription factors were evaluated at different ripening stages in the drupes of the Carolea and Tondina cultivars, sampled at different altitudes in the Calabria region, Italy. During drupe ripening, the total anthocyanin content and the transcript levels of analyzed genes gradually increased. In line with the anthocyanin content, a different level of expression of anthocyanin structural genes was observed in ‘Carolea’ compared to ‘Tondina’, and in relation to the cultivation area. Furthermore, we identified Oeu050989.1 as a putative R2R3-MYB involved in the regulation of anthocyanin structural genes correlated with the environmental temperature change response. We conclude that anthocyanin accumulation is strongly regulated by development, genotype, and also by environmental factors such as temperature, associated with the altitude gradient. The obtained results contribute to reducing the current information gap regarding the molecular mechanisms on anthocyanin biosynthesis regulation related to the environmental conditions in *Olea europaea*.

## 1. Introduction

The olive (*Olea europaea* L. subsp. *europaea* var. *europaea*) is one of the most iconic trees of the Mediterranean basin, with important implications from a social, economic, and ecological point of view [1]. However, olive tree cultivation is widespread all over the world, from temperate areas in the north to sub-tropical regions, and from low to high altitudes [2].

The olive tree produces edible fruits, classified as drupes, which are used for olive oil and olive table production. The physiological processes and metabolic pathways involved in drupes development, from growth to maturation and ripening, have been largely investigated and elucidated, given the agronomic relevance of olives [3,4].

During ripening, the olive drupes undergo various changes in their physiology, such as color alterations resulting from variations in pigment compounds (chlorophyll, flavonoids, and carotenoids) and texture modifications. There are also changes in turgor or cell wall composition, and modifications in the production of carbohydrates and other organic compounds, which alter organoleptic properties (nutritional value, aroma, and flavor) [5]. In addition, the expression of ripening-related genes and proteins are responsible for the ripening of olives, and they act under the control of a network of metabolic pathways that are in turn triggered by external and/or internal factors [6]. Environmental factors such as temperature, solar radiation, water stress, and soil can affect the anthocyanins content in fruits, altering both the qualitative and quantitative accumulations of antioxidant compounds [7,8,9].

Anthocyanins are water-soluble flavonoids, a class of secondary metabolites that are responsible for the red, purple, and blue colorations of flowers, fruits, and seeds. They act as visual signals to pollinators, as well as seed distributors [10]. In addition, they protect against biotic and abiotic stresses [11]. Furthermore, anthocyanins exert potential benefits in the human diet, including antioxidation, anti-inflammation, anti-aging, lowering lipids, anti-cancer, and neuroprotection [12,13].

Anthocyanins typically accumulate in plant vacuoles, and their biosynthesis occurs through the phenylpropanoid metabolic pathway. Anthocyanins synthesis is mainly controlled by two classes of genes, the anthocyanin biosynthesis structural genes, which encode the enzymes of the anthocyanin biosynthesis pathway, and the regulatory ones. The regulatory genes are transcription factors (TFs), mainly MYB (myeloblastosis), bHLH (basic helix-loop-helix) and WD40 (WD-40 with a scaffolding function) [14,15,16]. MYB TFs play a pivotal role in regulating the anthocyanin pathway [17], and can act alone or combined with bHLH and WD40 to form the ternary complex MYB-bHLH-WD40 (MBW) to regulate the structural genes of the anthocyanin pathway.

The structural genes (enzyme genes), involved in the anthocyanin biosynthetic pathway are well characterized and include phenylalanine ammonia lyase (PAL), cinnamate 4-hydroxylase (C4H), 4-cinnamate-CoA ligase (4CL), chalcone synthase (CHS) (which catalyzes the key reaction involved in flavonoid biosynthesis), flavanone 3-hydroxylase (F3H), and dihydroflavonol 4-reductase (DFR) (which synthesizes colorless leucoanthocyanidins). The final steps include anthocyanidin synthase (ANS) and flavonoid 3-O-glucosyltransferase (UFGT) that transform the colorless dihydrogen flavonols into colored anthocyanins [18,19,20,21,22].

Transcription profiling, performed through an Illumina RNA-seq approach on ‘Leucocarpa’ and ‘Cassanese’ drupes collected at different stages of maturation, was used to obtain information on the genes involved in the flavonoid and anthocyanin biosynthetic pathways during olive fruit ripening [23]. Furthermore, it was observed that some TF members with similarity to the MYB, MIC, and WD40 families were directly related to anthocyanin accumulation [23]. In addition, in the cv Leccino, the expression of anthocyanin biosynthesis structural genes was regulated by the above mentioned three TF families in both fruits and leaves [24].

The interaction between the TFs and the structural genes can promote or reduce anthocyanin accumulation through positive and negative regulation, respectively [25,26,27]. Among the MYB TFs family, the R2R3-MYBs type plays crucial roles as positive transcriptional regulators in anthocyanin production. For instance, in *Arabidopsis*, AtMYB75 (PAP1, At1g56650), AtMYB90 (PAP2, At1g66390), AtMYB113 (At1g66370), and AtMYB114 (At1g66380) of subgroup 6 promote anthocyanin biosynthesis in vegetative tissues by transcriptionally upregulating the expression of the structural genes [17].

*O. europaea* drupes collected from plants grown in areas at different altitudes, and therefore experiencing different climatic conditions, showed a modulation in the pathways of fatty acids as well as phenolic compounds in relation to both the drupe ripening stage and the growing area [28].

Despite this information, the molecular mechanisms involved in the regulation of anthocyanin biosynthesis in relation to the environmental conditions are still poorly known in *O. europaea*.

Starting from this scenario, we aimed to elucidate the effect of altitude on anthocyanin biosynthesis during the drupe development of two *O. europaea* cultivars, namely Carolea and Tondina, grown in the Calabria region (Italy). Attention was focused on changes in anthocyanin accumulation during drupe development, related to the three different cultivation areas. To achieve this goal, the expression levels of the genes encoding PAL, 4CL, CHS, CHI, F3H, flavonol 3′-hydrogenase (F3′H), flavonol 3′5′-hydrogenase (F3′5′H), DFR, ANS, and UFGT were evaluated through qRT-PCR. Moreover, in order to identify putative R2R3-MYB TFs, the selection, classification, and phylogenetic analysis of the R2R3-MYB gene family was performed in the oleaster (*O. europaea* var *sylvestris*) genome.

## 2. Results

### 2.1. Temperature Detection

**Figure 1:**
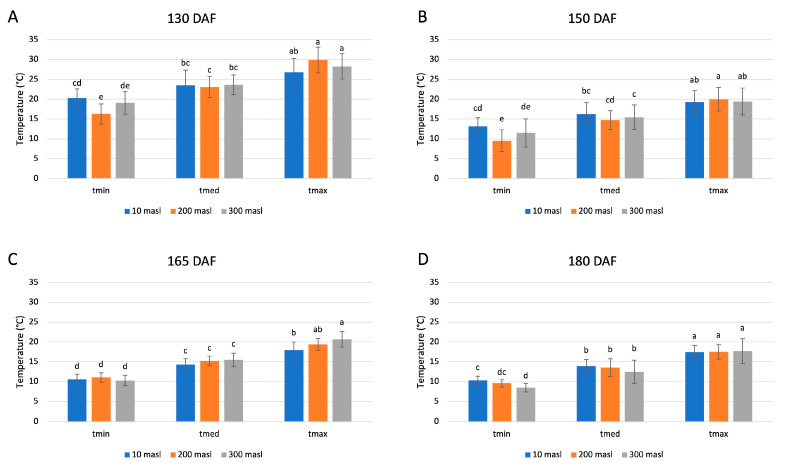
Minimum, medium, and maximum temperature values calculated as the average of the respective temperatures recorded over 15 days prior to the sampling performed at 130 (**A**), 150 (**B**), 165 (**C**), and 180 (**D**) days after flowering (DAF), at altitudes of 10, 200, and 300 m above sea level (masl). Different letters denote significant differences between the ripening stage and the growing area.

The minimum, medium, and maximum temperature values represent the average of the respective temperatures, recorded over 15 days prior to each sampling (Figure 1). The temperatures were measured in the three different areas under study, and four samplings.

The obtained data showed no significant differences among the three sites in the same considered temperature range and at each stage of the drupes ripening. Instead, significant changes were observed between the minimum and maximum temperature values collected at each stage of drupe ripening, and for each altitude considered. The highest temperature values were recorded at 130 days after flowering (DAF) (Figure 1A) and gradually reduced during drupes ripening. The lowest minimum temperature was 8.4 °C, reached at 300 masl of altitude at 180 DAF (Figure 1D), whereas the highest maximum temperature was 29.8 °C at 200 masl of altitude in drupes collected at 130 DAF (Figure 1A).

### 2.2. Total Anthocyanins

**Figure 2:**
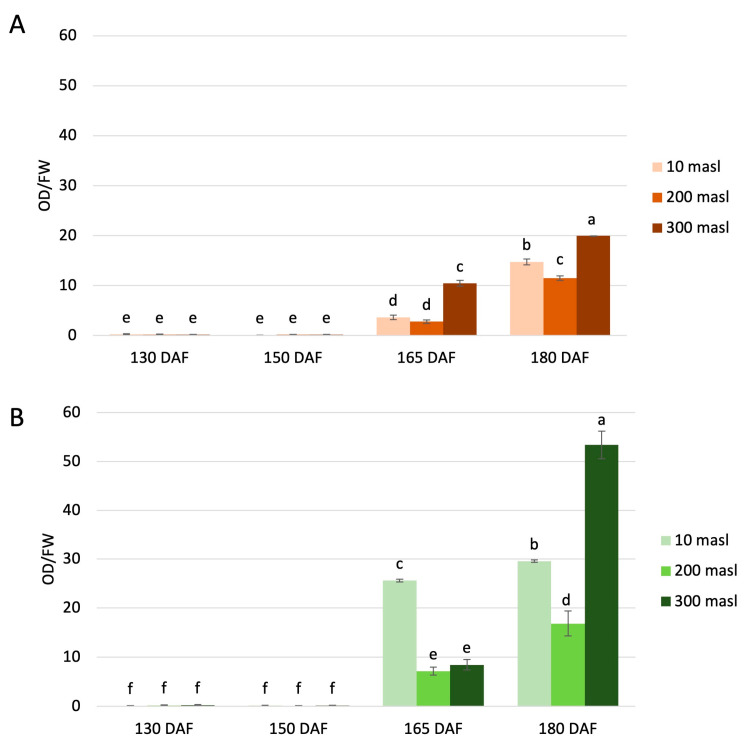
Quantification of total anthocyanins content in the Carolea (**A**) and Tondina (**B**) cultivars. Analysis was performed in drupes harvested at 130, 150, 165, and 180 days after flowering (DAF) from plants grown at different meters above sea level (masl). The results were reported as the mean value (±standard deviation) of three replicas. Statistical analyses were performed using one-way ANOVA with the Tukey post hoc test (*p* < 0.05) after the Shapiro-Wilk normality test. Different letters denote a significant difference between the ripening stage and the growing area.

The UV spectrophotometric determination of the total anthocyanin levels was carried out in the Carolea and Tondina cultivars at each stage considered (Figure 2). The anthocyanin content was significantly different between the drupes, both in relation to the maturation stage and the altitude. Differences were also observed in relation to the genotype. Indeed, the total amount of anthocyanins was higher in ‘Tondina’ than ‘Carolea’. Our results showed a very low content of total anthocyanins in drupes collected at 130 and 150 DAF compared to the drupes sampled at the other stages of ripening. An increase in anthocyanin amount was observed starting from 165 DAF, closely related to the development of the purple coloration of the fruit, in both the Carolea (Figure 2A) and Tondina cultivars (Figure 2B).

In ‘Carolea’, comparing the drupes collected at 180 DAF vs. 165 DAF, the anthocyanins content increased above four-fold at 10 masl and 200 masl, and two-fold at 300 masl, although the highest quantity is always present at 300 masl (Figure 2A).

With respect to ‘Tondina’ at 165 DAF, the highest anthocyanin content was measured at 10 masl of altitude, which was about 3.6- and 3-fold higher than the values measured at 200 and 300 masl, respectively. When comparing the drupes at 180 DAF vs. 165 DAF, a slow increase was recorded in the drupes harvested at 10 masl, while a significant increase of about 2- and 6.3-fold was monitored in drupes grown at 200 and 300 masl, respectively (Figure 2B).

### 2.3. Expression Profiles of Anthocyanin Biosynthesis-Related Genes

**Figure 3:**
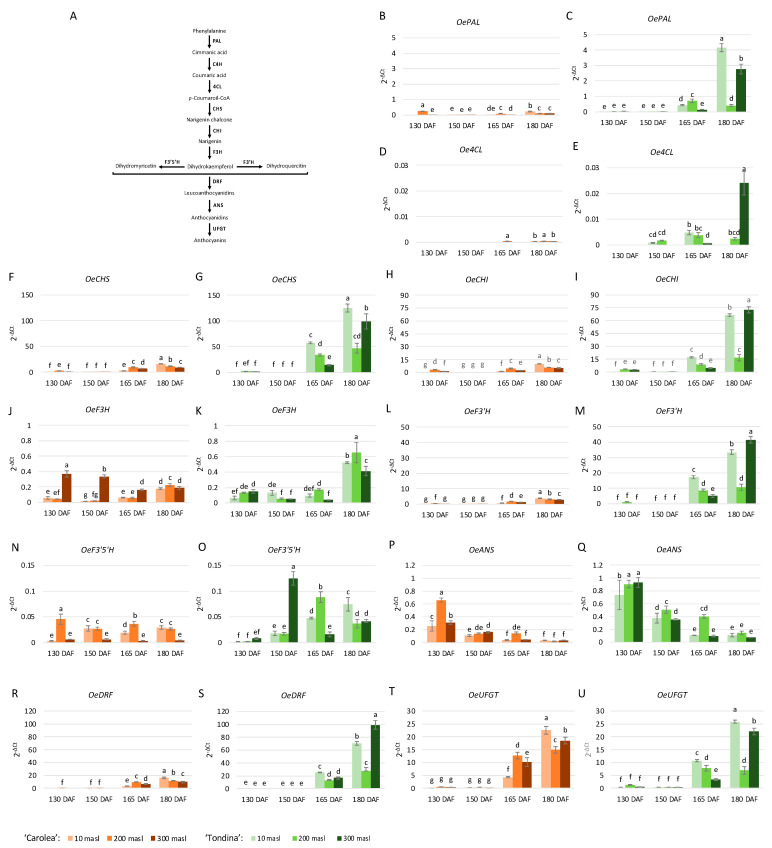
Schematic representation of the main step in the anthocyanin biosynthetic pathway (**A**). Phenylalanine ammonia lyase (PAL), cinnamate 4-hydroxylase (C4H), 4-coumarate-CoA ligase (4CL), chalcone synthase (CHS), chalcone isomerase (CHI), flavonol 3-hydrogenase (F3H), flavonol 3′-hydrogenase (F3′H), flavonol 3′5 ′-hydrogenase (F3′5′H), dihydroflavonol 4-reductase (DFR), anthocyanidin synthase (ANS), and UDP-glucose:anthocianidin:flavonoid glucosyltransferase (UFGT). Relative expression levels of *OePAL*, *Oe4CL*, *OeCHS*, *OeCHI*, *OeF3H*, *OeF3′H*, *OeF3′5′H*, *OeDFR*, *OeANS*, and *OeUFGT* genes (**B**–**U**) in ‘Carolea’ (orange scales) and ‘Tondina’ (green scales) drupes harvested at 130, 150, 165, and 180 days after flowering (DAF) from plants grown at different meters above sea level (masl). The results were reported as mean value (±standard deviation) of three replicas. Statistical analyses were performed using one-way ANOVA with the Tukey post hoc test (*p* < 0.05) after the Shapiro-Wilk normality test. Different letters denote significant differences between ripening stage and growing area.

In addition to the anthocyanin content, the transcript abundance of anthocyanin-related genes was evaluated via qRT-PCR (Figure 3). The obtained data indicate that the considered genes were differentially expressed in the ‘Carolea’ and ‘Tondina’ drupes with respect to ripening stage, altitude, and genotype.

A general upregulation of the anthocyanin genetic pathway was observed in ‘Tondina’ drupes compared to ‘Carolea’ ones.

Concerning the ‘Carolea’ cultivar, *OePAL*, expression increased during ripening in drupes grown at 10 and 300 masl, reaching the highest values at 180 DAF. On the contrary, *OePAL* expression showed an opposite trend at 200 masl (Figure 3B). *Oe4CL* was expressed, at low levels, at the last stage of ripening in all drupes of the studied populations, with the exception of the drupes grown at 200 masl. In this case, the expression levels were detected starting from 165 DAF, and were similar to those observed at 180 DAF. With respect to *OeCHS*, *OeCHI*, and *OeF3′H*, globally, they showed a similar expression trend during ripening. A gradual upregulation during the time-course occurred, even if the expression levels were higher at 130 than 150 DAF (Figure 3F,H,L). *OeF3H* resulted in a gradual upregulation in the above-described genes, except in the populations growing at 300 masl, in which it was gradually downregulated. *OeF3’5’H* showed a different expression trend with respect to altitude. In the drupes harvested at 10 masl, a low *OeF3’5’H* expression level was found at 130 DAF, and this increased approximately 10-fold at 150 and 180 DAF, and 7-fold at 165 DAF (Figure 3N). An opposite behavior was observed in the drupes sampled at 200 masl. Indeed, the *OeF3’5’H* levels were higher at 130 and 165 DAF than at 150 and 180 DAF. In the fruits collected at 300 masl, a low and similar expression of *OeF3’5’H* was observed (Figure 3N). *OeANS* was gradually downregulated over the time in the drupes of all the analyzed populations (Figure 3P). Finally, *OeDRF* and *OeUFGT* showed a similar expression pattern, reaching the highest transcription values in the last two ripening stages analyzed (Figure 3R,T).

Considering the ‘Tondina’ genotype, a general upregulation of the anthocyanin biosynthesis-related genes was observed during the last two ripening stages, corresponding to the onset of color change. Likewise, for that observed in ‘Carolea’, *OeANS* showed a gradual downregulation during drupe ripening (Figure 3Q). Nonetheless, some genes exhibit a peculiar form of expression during the time-course. *Oe4CL* was expressed starting from 150 DAF, and this increased to 165 DAF and was no longer expressed at 180 DAF in the drupes harvested at 10 masl; it had a bell-trend from 150 to 180 DAF in the drupes sampled at 200 masl. Whereas, it was poorly expressed at 165 DAF in the fruits harvested at 300 masl, and it had the highest expression values at 180 DAF (Figure 3E). The *OeCHS* and *OeUFGT* genes showed the same expression trend in which an increase in their expression during the time-course with a greater upregulation in the drupes collected at 10 masl at 165 and 180 DAF was observed (Figure 3G,U). The transcription of the *OePAL*, *OeCHI*, *OeF3’H*, and *OeDRF* genes increased at 165 DAF and at 180 DAF, in which, respectively, the drupes harvested at 10 masl and at 300 masl showed the highest level (Figure 3C,I,M,S). *OeF3H* was expressed during the time-course in the drupes harvested at 10, 200, and 300 masl; and upregulated in the samples collected at 180 DAF (Figure 3K). Similar to that observed in ‘Carolea’, *OeF3’5’H*, showed a different expression trend based on the altitude at which the drupes were harvested. A gradual increase in expression level was observed at 10 masl during the time-course. *OeF3’5’H* expression increased from 130 DAF to 165 DAF and decreased to 180 DAF at 200 masl, and finally, the transcription levels peaked at 150 DAF, decreased at 165 DAF, and then increased to 180 DAF in drupes collected at 300 masl (Figure 3O).

### 2.4. Selection, Classification, and Phylogenetic Analysis of the R2R3-MYB Gene Family in Olea europaea var sylvestris

According to the number of domains observed in each gene, the 217 genes retrieved in PlantTFDB were assigned to three subfamilies: 13 R1-MYB members (containing one repeat), 190 R2R3-MYB members (containing two repeats), 7 R1R2R3-MYB (containing three repeats), and 2 atypical-MYB genes (containing 4 repeats) (Supplementary Table S1). The remaining seven genes did not have the PF00249 consensus pattern (Supplementary Table S1).

**Figure 4:**
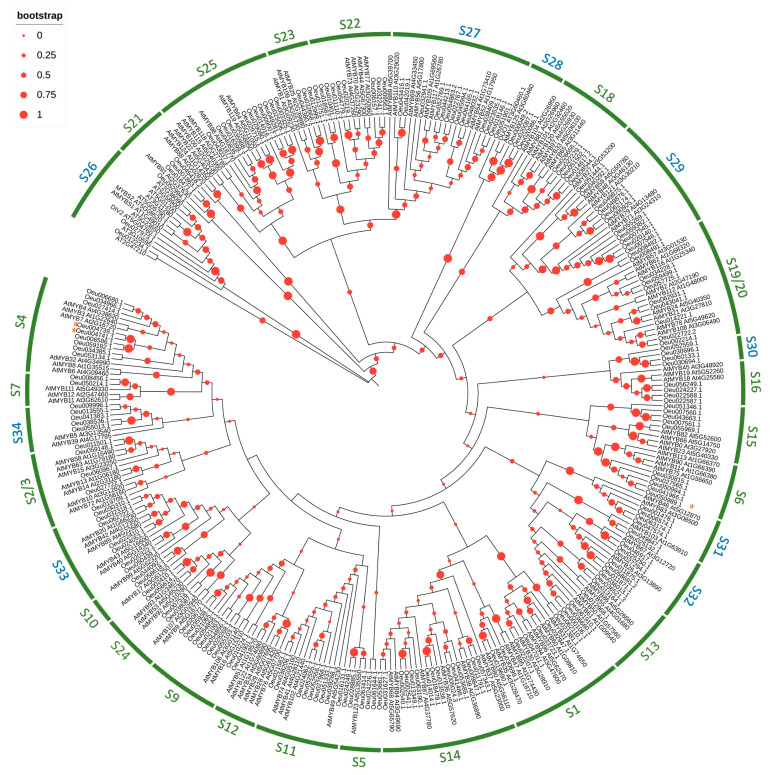
Phylogenetic tree of the *Olea* and *Arabidopsis* R2R3-MYB protein sequences. The evolutionary history was inferred using the neighbor-joining method. The complete amino acid sequences of 190 *Oe*R2R3-MYB and 138 *At*R2R3-MYB proteins were used. The subgroups S1 to S25, named according to the previous principle [17], were marked in green, whereas the remaining subgroups, named S26 to S34, were marked in blue.

A neighbor-joining tree was generated based on multiple alignments of the predicted amino acid sequences of the R2R3-MYB domains from *O. europaea* var *sylvestris* and *Arabidopsis* (Figure 4) to understand the evolutionary relationships between OeR2R3-MYB proteins, and known R2R3-MYB proteins from *Arabidopsis*. This tree could also give us insights to predict gene classifications and functions.

Using a sequence-similarity topology and subgroup classification previously reported [17], all R2R3-MYB members from *Olea* and *Arabidopsis* were classified into 30 subgroups, of which the subgroups S1 to S25 were named according to the previous principle [17], and the remaining subgroups were named S26 to S34 (Figure 4). Furthermore, the *Arabidopsis* sequences previously contained in S2 and S3, as well as those in S19 and S20, were found clustered in a single subgroup named S2–S3 and S19–S20, respectively.

Notably, except for S30, all subgroups were clustered members from the two species, indicating that the expansion of the R2R3-MYB members may have occurred before the divergence of the O. europaea plants and Arabidopsis. The subgroup S30 contained only Olea sequences; nevertheless, this was close to S16, suggesting a recent gene duplication, or one subsequent to the separation of the two species, could have occurred between the members of the two subgroups.

In previous studies, *AtMYB75*, *AtMYB90*, *AtMYB113*, and *AtMYB114* of S6 have been designated to participate in the phenylpropanoid pathway/anthocyanin biosynthesis [29,30]. In addition, *AtMYB4* of S4 was reported as a MYB repressor in the anthocyanin pathway [31]. Based on phylogenetic and classification analysis, we investigated the expression levels of *Oeu050989.1* (retrieved in the S6 subgroup), as well as *Oeu004741.1* and *Oeu004739.1* (both present in the S4 subgroup), in both cultivars at the different ripening stages.

**Figure 5:**
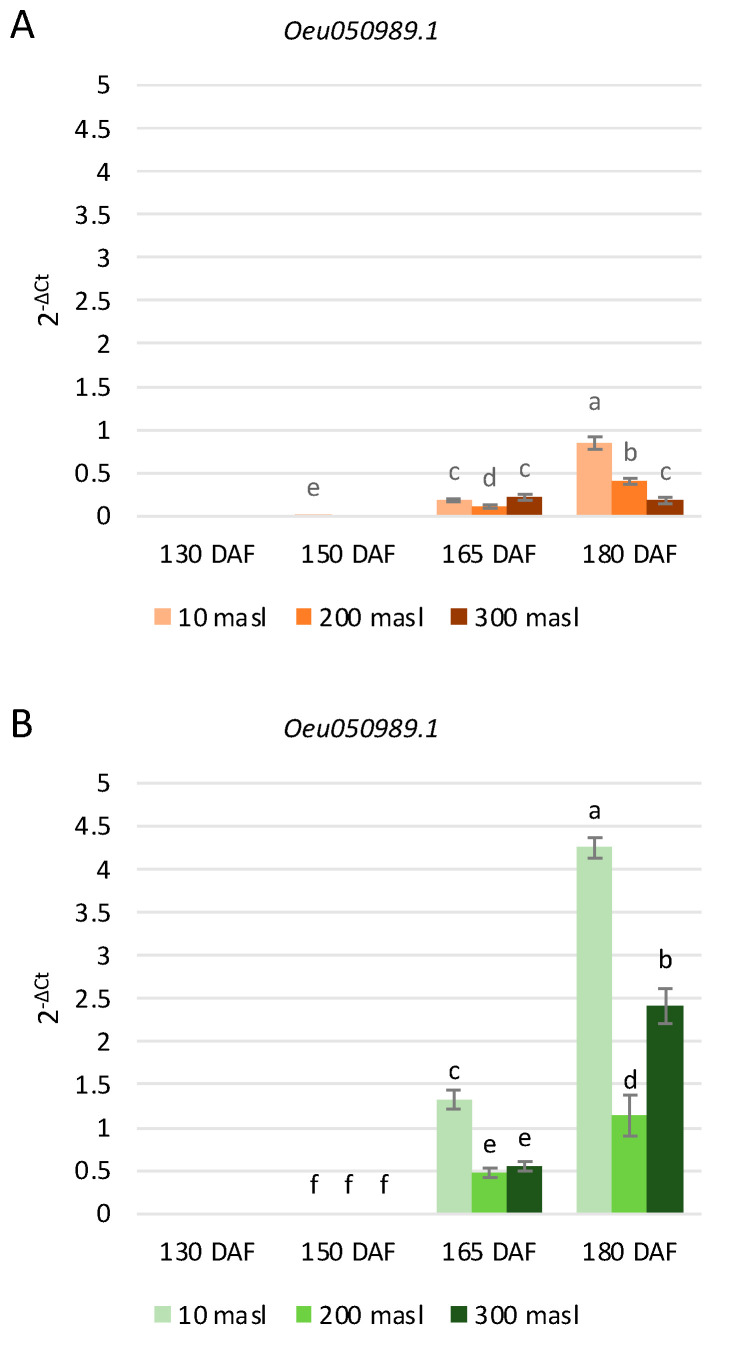
Relative expression levels of *Oeu050989.1* in ‘Carolea’ (**A**) and ‘Tondina’ (**B**). Analyses were performed on drupes harvested at 130, 150, 165, and 180 days after flowering (DAF), from plants grown at different meters above sea level (masl). Statistical analyses were performed using one-way ANOVA with the Tukey post hoc test (*p* < 0.05) after the Shapiro-Wilk normality test. Different letters denote significant differences between ripening stage and growing area.

The *Oeu050989.1* gene showed a higher expression level in ‘Tondina’ than in ‘Carolea’ (Figure 5), and it was mainly expressed at 165 and 180 DAF, corresponding to the onset of color change. No expression levels were detected at 130 DAF in both of the studied cultivars (Figure 5). With respect to ‘Carolea’, the *Oeu050989.1* expression level increased in drupes collected at 10 and 200 masl, starting from the 165 DAF to 180 DAF stages of ripening, whereas no changes were detected in fruits harvested at 300 masl (Figure 5A). In ‘Tondina’, a gradual upregulation of this gene was observed from 150 to 180 DAF at all altitudes condidered. (Figure 5B).

**Figure 6:**
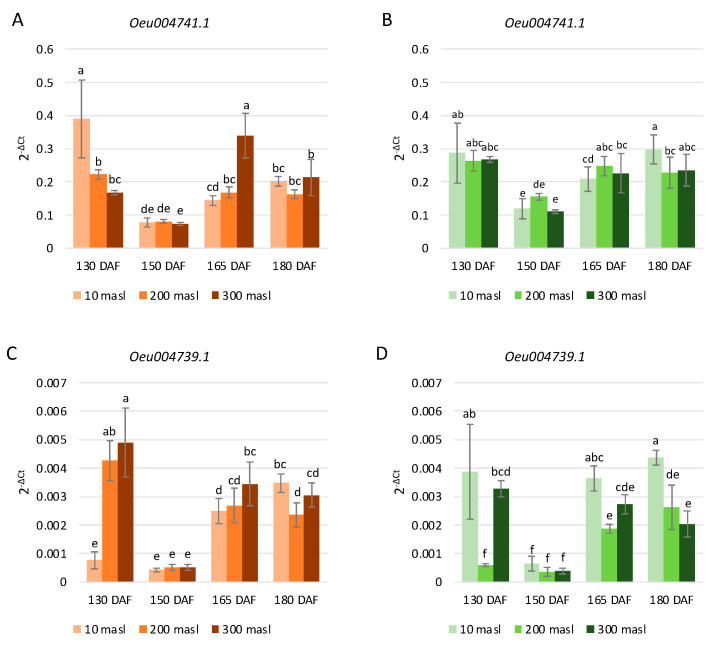
Relative expression levels of *Oeu004741.1* (**A**,**B**) and *Oeu004739.1* (**C**,**D**) in ‘Carolea’ (**A**,**C**) and ‘Tondina’ (**B**,**D**). Analyses were performed on drupes harvested at 130, 150, 165, and 180 days after flowering (DAF) from plants grown at different meters above sea level (masl). Statistical analyses were performed using one-way ANOVA with the Tukey post hoc test (*p* < 0.05) after the Shapiro-Wilk normality test. Different letters denote significant differences between the ripening stage and growing area.

Concerning the selected genes belonging to the S4 subgroup, in ‘Carolea’ and ‘Tondina’, a different trend of their expression was observed, related to both the altitude and ripening stages. In the population grown at 10 masl, *Oeu004741.1* and *Oeu004739.1* expression were decreased at 150 vs. 130 DAF and then increased progressively at 165 and 180 DAF in both cultivars (Figure 6A–D). In fruits collected at 200 masl, the gene transcription level of both *Oeu004741.1* and *Oeu004739.1* decreased from 130 to 150 DAF, then increased at 165 DAF and remained substantially stable until 180 DAF in both genotypes (Figure 6A–D). Finally, in drupes harvested at 300 masl, the *Oeu004741.1* and *Oeu004739.1* gene expression levels were reduced at 150 vs. 130 DAF and increased at 165 DAF. No changes were detected in both the S4 subgroup selected genes levels at 180 vs. 165 DAF with the exception of cv Carolea drupes, in which we observed a significant decrease (Figure 6A).

Furthermore, in both cultivars, the transcription levels of *Oeu004739.1* were dramatically lower than in *Oeu004741.1* (Figure 6).

**Figure 7:**
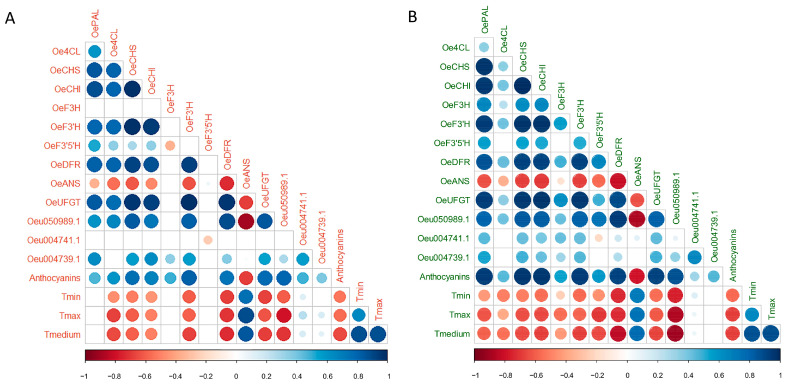
Correlation analysis among *OePAL*, *Oe4CL*, *OeCHS*, *OeCHI*, *OeF3H*, *OeF3′H*, *OeF3′5′H*, *OeDFR*, *OeANS*, *OeUFGT*, *Oeu050989.1*, *Oeu004741.1*, and *Oeu004739.1* genes; and total anthocyanin content and temperatures in ‘Carolea’ (**A**) and ‘Tondina’ (**B**). Analyses were performed using the Pearson correlation and correlation coefficients *r* > 0.70, and the corresponding *p*-values < 0.05 were used as the cut-off threshold. Red color indicates negative correlation (−1); blue color indicates positive correlation (+1); and the color intensity represents the strength of correlation.

We performed correlation analyses between gene expression, total anthocyanin content, and temperatures in both ‘Carolea’ and ‘Tondina’ (Figure 7). According to the screening criteria (correlation coefficient r > 0.7, *p* < 0.05), in both ‘Carolea’ and ‘Tondina’, *OeDRF* and *Oeu050989.1* expression were both significantly and negatively correlated with the temperature changes (Figure 7 and Supplementary Table S2). Moreover, the transcription levels of *Oeu050989.1* were positively correlated to *OeCSH*, *OeCHI*, *OeF3′H*, *OeDRF*, and *OeUFGT* levels in ‘Carolea’ drupes (Figure 7). In addition, *OePAL* and *OeF3′5′H* transcription levels were also significantly correlated with *Oeu050989.1* expression in ‘Tondina’ (Figure 7B and Supplementary Table S2).

Conversely, no correlation was found between the expression levels of *Oeu004741.1* and *Oeu004739.1* (belonging to the S4 subgroup), and anthocyanins biosynthesis-related genes, and neither between their expression levels and anthocyanin content, for both the Carolea and Tondina cultivars.

Finally, total anthocyanin content was significantly correlated with the temperature medium values in both cultivars, with a correlation coefficient of −0.66 and −0.69 in ‘Carolea’ and ‘Tondina’, respectively (Figure 7 and Supplementary Table S2). In addition, the total anthocyanins content was significantly and positively correlated with the expression levels of the *OeCSH*, *OeF3′H*, *OeDRF*, *OeUFGT*, and *Oeu050989.1* genes in both cultivars.

## 3. Discussion

In the present work, we assayed the anthocyanin amount and the expression levels of the transcripts along the anthocyanin biosynthetic pathway by comparing the fruits of two olive cultivars collected at different stages of ripening. Therefore, drupes were collected at 130, 150, 165, and 180 DAF, corresponding to the ripening stages in which the transition from green mature to turning purple stages occurs.

Previously, we reported that *O. europaea* drupes showed differential modulation for both the fatty acids and secondary metabolites pathways, i.e., phenols, in relation to altitude [28]. Thus, we verified whether and how anthocyanin biosynthesis differs in relation to the altitude.

In olive, a large variation in the phenolic composition was observed, including an increase in anthocyanins corresponding to the ripening trends of fruits [32]. During the ripening of drupes, the biosynthesis of anthocyanins begins when oleuropein decreases as a direct consequence of an increase in the enzymatic activity of phenylalanine-ammonium lyase (PAL) [32]. Interestingly, a reduction in the oleuropein content between 150 and 180 DAF was found in the ‘Carolea’ drupes collected at both 10 and 200 masl [28].

Our results revealed a very low content of total anthocyanins in drupes harvested at 130 and 150 DAF in all three grown areas, in both ‘Carolea’ and ‘Tondina’. Furthermore, a gradual increase in the anthocyanins amount was observed between the sampling dates, a trend closely related to the development of the purple drupes color. Moreover, the different altitude at which olive fruits ripened impacted the accumulation of the total content of anthocyanins. Indeed, the higher anthocyanin levels were measured at 300 masl at the lowest minimum temperature. Nevertheless, other factors can affect the anthocyanin accumulation.

In blueberries, the low daily temperatures at higher altitudes, as well as the sunlight spectra, visible light, and UV radiation, also seem to correlate with the accumulation of anthocyanin [33,34]. Furthermore, high temperatures decrease anthocyanin content in the skins of apples and grapevine berries [35,36].

Our data showed that several genes related to anthocyanin biosynthesis in both *O. europaea* ‘Carolea’ and ‘Tondina’ reflected the quantities of total anthocyanins content and were associated with both fruit ripening and the environmental factors to which they were subjected to.

Previous data have shown that in *O. europaea* cv Leccino, the concentration of flavonoids increased in young fruits, and anthocyanins were accumulated during ripening, in particular in the epicarp, in conjunction with the upregulation of the *PAL*, *CHS*, *F3H*, and *UFGT* genes [21]. The *DFR* gene was induced in the epicarp at the beginning of the drupe color change, whereas *ANS* were mainly expressed at a more advanced stage [21].

Transcriptomic analysis on *O. europaea* cv Leccino revealed the presence of nine *ANS/LDOX* genes that were differentially expressed during fruit ripening [24]. Some of these isoforms were up- or downregulated during the later stages of maturation (140 and 170 DAF), while some exhibited a bell-like expression trend, from 50 to 170 DAF [24].

In our case, the gradual *OeANS* downregulation observed in both ‘Carolea’ and ‘Tondina’ may be due to the isoform that tends to downregulate during maturation.

With respect to *OeF3’H* and *OeF3’5’H* genes, we observed a different expression trend related to the altitude at which the drupes were harvested. However, at the post-transcriptional level, a different kind of regulation occurs, and our results can suggest a different content of dihydroquercetin and dihydromyricetin from which different anthocyanins are synthesized in response to different pedoclimatic conditions.

Dihydrokaempferol is an important precursor of anthocyanin biosynthesis that can be hydroxylated by both the flavonoid 3′-hydroxylase (F3′H) and flavonoid 3′, 5′-hydroxylase (F3′5′H) into dihydroquercetin and dihydromyricetin, respectively. These two enzymes are responsible for the structural diversity of anthocyanins and influence their B-ring hydroxylation patterns and their colors [37].

In grape berries (*Vitis vinifera*), the transcription of *VvF3′H* and *VvF3′5′H* was related to flavonoids composition [8] and different abiotic stresses, such as shading, water stress, and low temperatures, causing a different expression of these genes, and as a consequence, a different content of di- and tri-substituted flavonoids [38,39,40]. Indeed, the exposure of the bunch to the sun increased both the proportion of B ring trihydroxylation in anthocyanins and the expression of *VvF3′5′H* [39]. Furthermore, high temperatures affect the relative proportion of di- and tri-substituted flavonoids in the berry by reducing the expression ratio between *VvF3′5′H* and *VvF3′H*, and the proportions of proanthocyanidins and tri-substituted anthocyanins [40]. In addition, water stress induced the gene expression of *VvF3′5′H*, with a significant increase in 3′4′5′-hydroxylated anthocyanins [38].

In ‘Carolea’ a generally lower expression level of *F3′H* and *F3′5′H* genes than in ‘Tondina’ was observed during the analyzed ripening stages, in agreement with the quantified anthocyanin content. Thus, the observed differences in anthocyanin levels in ‘Carolea’ vs. ‘Tondina’ were mainly related to the differences in the expression levels of the related genes. Moreover, it is possible to hypothesize a different degree of sensitivity and/or acclimatization to the pedoclimatic conditions shown by the cultivars analyzed.

The anthocyanin biosynthesis pathway is controlled by TFs with similarity to the MYB, MIC, and WD40 family [41,42,43], which form the ternary complex MBW and play a key role in the accumulation and inhibition of anthocyanin and proanthocyanin. In *O. europaea* cv Cassanese, as well as in cv Leccino, different transcription factor members were found to be involved in anthocyanin biosynthesis [23,24].

In the present work, a higher number of R2R3-MYB members (190) were identified in *O. europaea* var *sylvestris* than other species—for instance, *Camellia sinensis* (122) [44], *Solanum tuberosum* (124) [45], *Hypernicum perforatum* (109) [46], and *Ananas comosus* (94) [47]. This result implied that R2R3-MYB gene replication occurred widely in species evolution and played a crucial role in the regulation of specific traits of plant development [48].

A neighbor-joining tree was constructed from multiple alignments of the R2R3-MYB protein sequences from *Arabidopsis* and *Olea* to investigate evolutionary relationships. The analytical results led to the separation of the R2R3-MYB proteins into 31 subgroups. Notably, the subgroups were clustered members from the two species, indicating that the expansion of the R2R3-MYB members may have occurred before the divergence of the *O. europaea* plants and *Arabidopsis*, with the exception of the S30 subgroup. This subgroup contained exclusively *Olea* sequences, indicating a likely derivation from a duplication that occurred after the divergence of the *Olea* and *Arabidopsis* during their evolutionary history.

It is supposed that members belonging to the same branch may experience a common evolutionary process and possess a conserved function. This study compared the R2R3-MYB genes of *Olea* and *Arabidopsis*, elucidating the landscape of *Olea* R2R3-MYB preservation and amplification. The phylogenetic tree of R2R3-MYB protein also contributes to predicting the function of the *Olea* R2R3-MYB protein based on the reported branches of *Arabidopsis thaliana* MYBs. Our attention was focused on genes related to anthocyanin biosynthesis. For example, in the phylogenetic tree, the MYB TFs of the S6 and S4 subgroups might be regulators of the phenylpropanoid pathway [29,30,31].

In the present study, the *Oeu050989.1* gene, belonging to the S6 subgroup, was mainly expressed at 165 and 180 DAF, corresponding to the onset of fruit color change. The expression of this gene was positively correlated with those of *OeCSH*, *OeCHI*, *OeF3′H*, *OeDRF*, and *OeUFGT* in both the Carolea and Tondina cultivars. Interestingly, the expression of *Oeu050989.1* and *OeDRF* was negatively correlated with the environmental temperature trend, while the remaining analyzed genes did not show a significant correlation. These results confirm that *OeDRF* play a key role in the regulation of anthocyanin biosynthesis in olives [21], and suggest that *Oeu050989.1* could be a crucial role as a transcriptional regulator in the production of anthocyanins in the temperature changes response.

On the other hand, AtMYB4-like type repressors (belong to S4 subgroup) act as direct repressors by binding to the MYB motifs found in the promoters of many structural genes in the anthocyanin pathway, leading to the downregulation of this pathway [27]. The suppression of *ANS*, *DFR*, and *UFGT* has been a common feature observed in all transgenic tobacco and other plants heterologously expressing the AtMYB4-like repressor [49,50,51].

In the present study, no association between the expression levels of *Oeu004741.1* and *Oeu004739.1*, and anthocyanin biosynthesis-related genes was found, nor between the expression levels of both genes and the drupes anthocyanin content, suggesting that these genes are not involved in the regulation of anthocyanin pigmentation in *Olea europaea* cv Carolea and Tondina.

## 4. Materials and Methods

### 4.1. Plant Material

Drupes of ten/eleven-year-old plants of both *Olea europaea* Carolea and Tondina cultivars were collected from clonal populations belonging to the olive germplasm collections of CREA-OFA at Mirto Crosia (Cosenza, Calabria, Italy, latitude 39°37′04.57″N; longitude 16°45′42.00″ E, altitude 10 masl), at Rende (Cosenza, Calabria, Italy, latitude 39°21′58″ N; longitude 16°13′44″ E, altitude 200 masl) and from a farm located at Terranova da Sibari (Cosenza, Calabria, Italy, latitude 39°40’50” N; longitude 16°22’03” E, altitude 300 masl).

The same agronomic practices without irrigation were used for all populations, and sampling was performed in the seasons of 2019/2020 and 2020/2021 at 130, 150, 165, and 180 days after flowering (DAF). A total of 20 drupes were harvested from 5 plants grown in the same farm (*n* = 100 drupes for each sampling). For each stage of maturation, the drupes were pulled together to minimize individual variation and to reduce variability [52,53]. All samples were fixed in liquid nitrogen and stored at −80 °C for both total anthocyanin quantification and qRT-PCR experiments.

The environmental temperature was monitored with the ONSET HOBOware (HOBO, Onset Computer Corporation, Pocasset, MA, USA) experimental weather station, equipped with an air temperature sensor.

### 4.2. Total Anthocyanin Quantification

For each sample, total anthocyanins were extracted from a pool of epi-mesocarp drupes tissues (*n* = 20 × 5 plants). Samples were pulverizing in liquid nitrogen by using a mortar and pestle.

Briefly, 300 mg of pulverized material were incubated with 1 mL of acidic methanol (1% HCl, *w*/*v*) at 4 °C in the dark, overnight, according to [54]. The extracts were centrifuged at 10,000× *g* for 15 min at 4 °C. The clear supernatant was transferred into a new tube, and the anthocyanin content was determined at 530 nm and 657 nm using a Shimadzu UV-1601 spectrophotometer (Shimadzu, Kyoto, Japan). Total anthocyanin concentration was calculated according to [54] using the following formula:

$$Q_{Anthocyanins}={(A}_{530}-0.25A_{657})/FW$$

where *Q_Anthocyanins_* is the anthocyanins amount, *A_530_* and *A_657_* are the absorption values at the indicated wavelengths, and *FW* is the sample fresh weight. Each sample was analyzed in triplicate; the obtained data represent the mean ± SD of three independent experiments. Statistical analyses were performed using a one-way analysis of variance (ANOVA) followed by Tukey’s post hoc test (*p* < 0.05).

### 4.3. RNA Isolation and Real-Time Quantitative PCR Analysis

Total RNA was extracted from 100 mg of a pool of the epi-mesocarp tissues of drupes (*n* = 20 × 5 plants) harvested at 130, 150, 165, and 180 DAF, as previously described [28]. A total of 1 µg of total RNA for each sample was retro-transcribed into cDNA using a SuperScript III Reverse Transcriptase kit (Invitrogen, Waltham, MA, USA), according to the manufacturer’s protocol.

Primers used for the quantitative real-time PCR analysis (qRT-PCR) of anthocyanin biosynthesis-related genes were selected according to [21,23] (Table 1).

Primers used to quantify *Oeu050989.1*, *Oeu004741.1*, and *Oeu004739.1* MYB genes were designed using Primer Express™ Software v3.0.1 (Applied Biosystems, Foster City, CA, USA) and selected according to robustness, specificity, and consistency. Only the pairs with an average efficiency of between 0.90 and 1.0 were used (Table 1). Amplification reactions were performed using a STEP ONE instrument (Applied Biosystems, Foster City, CA, USA), and were prepared in a final volume of 20 μL containing: 1X Select SYBR^®^ Green PCR Master Mix (Applied Biosystems, Foster City, CA, USA), 0.2 μM of each primer, and 50 ng cDNA. The reactions were carried out in triplicate in 48-well reaction plates, and negative controls were included. The cycling parameters were as follows: 50 °C for 2 min and 95 °C for 2 min, followed by 40 cycles of 95 °C for 10 s and 60°C for 30 s. Melting curve analysis was also performed to confirm the existence of a unique PCR product, and evaluated via an increase of 0.5 °C every 10 s, from 60 °C to 95 °C.

The relative quantification of gene expression was calculated according to Schmittgen and Livak, 2008 [56]. The ΔCt values were obtained by calculating the difference of the Ct of each target, compared to the arithmetic mean of the Ct for the *OeCRY2* housekeeping gene [55] (Table 1), and the relative expression was expressed as 2^−ΔCt^ [57].

Statistical analyses were performed using ANOVA and a Tukey’s rank test (*p* < 0.05).

### 4.4. Identification and Phylogenetic Analysis of the O. europaea var sylvestris OeR2R3-MYB Proteins

With the aim of identifying the regulatory interaction between MYB transcription factors (TFs) and their target genes, the Plant Transcription Factor Database (PlantTFDB; http://planttfdb.cbi.pku.edu.cn, accessed on 20 July 2022) was used as a high-quality resource [58].

Genes of *Arabidopsis thaliana* and *Olea europaea* var. *sylvestris* encoding MYB transcription factors were retrieved from PlantTFDB, whereas the MYB transcription factors sequences of olive cultivars were not available in the database.

The primary search disclosed 217 and 168 members annotated as “MYB” in the *Olea* and *Arabidopsis*, respectively, in the plantTFDB database. The presence of MYB-repetition domains (the PF00249 consensus pattern) in *Olea* protein sequences was checked with PROSITE (https://prosite.expasy.org/scanprosite/, accessed on 20 July 2022), with the aim of identifying the R2R3-type sequences. The protein sequences lacking the MYB-DNA binding domain, even if they were annotated as a MYB protein family in the plantTFDB database, were discarded.

The *Arabidopsis* R2R3-type sequences were selected according to [59]. The gene identifiers were assigned to each *AtMYB* gene to avoid confusion when multiple names were used for the same gene. Uncharacterized *AtMYB* genes were denoted by their locus ID.

The full-length amino acid sequences of 190 OeR2R3-MYB proteins (*Olea europaea* var *sylvestris*) and 138 AtR2R3-MYB proteins (*Arabidopsis thaliana*) were used to explore the corresponding phylogenetic relationships. The sequences were aligned, and a neighbor-joining (NJ) phylogenetic tree was constructed using the MEGA X program with the *p*-distance parameter [60]. A total of 1000 replicates were used for the pairwise deletion and bootstrap analysis.

### 4.5. Pearson Correlation Analysis

A Pearson correlation was used among the expression of analyzed genes, total anthocyanin content, and temperatures, in both ‘Carolea’ and ‘Tondina’. Pearson correlation coefficients (r) and the corresponding *p*-values (*p*) were calculated by using the Statistical Software R package “corrplot”, based on the criteria of r > 0.70 and *p* < 0.05.

## 5. Conclusions

Taken together, our results suggest that anthocyanin accumulation is strongly affected by both developmental stage and genotype in olive. The environmental temperature on anthocyanin content and structure-related genes in drupes is mainly associated with the ripening progression. The reduction in drupes pigmentation under high temperatures for ‘Carolea’ and ‘Tondina’ implies great challenges in olive growing in order to maintain the nutritional and antioxidant quality, taking into account the central role of these cultivars for Calabria olive growing. In addition, ‘Tondina’ drupes, used for both olive oil production and table olives, could represent a precious source of beneficial compounds for human health.

Finally, for the first time, the classification and phylogenetic analysis of the R2R3-MYB gene family in *Olea europaea* var *sylvestris* was conducted, and the gene expression analysis demonstrated that *Oeu050989.1* may be responsible for the transcription regulation of anthocyanin synthesis in drupes under a low temperature regime.

## Figures and Tables

**Table 1:** List of primers used for qRT-PCR analysis.

Gene	Forward	Reverse	Reference
*OePAL*	5’-ACACATCCATCTTCCAAAAG-3’	5’-GTTCCCAGTTCTTCCCTTAC-3’	[23]
*Oe4CL*	5’-AAATTTAAAGGCTTCCAGGT-3’	5’-GCTTCTTCGGTAAGTTCAAA-3’	[23]
*OeCHS*	5’-GATTGGAACTCGATTTTCTG-3’	5’-GGACTTTCTCATCTCATCCA-3’	[23]
*OeCHI*	5’-AGGGTTCACGTATGGAGTG-3’	5’-TGCAAATACAATCTCAGCAG-3’	[23]
*OeF3H*	5’-TCCTCTGCCCGTGTGATAGT-3’	5’-AATCCGTGTGATGCAGTGAG-3’	[23]
*OeF3’H*	5’-GTGGCAGAAGCTGACCTACC-3’	5’-CGTAGAGCCCTTTGGAATGA-3’	[23]
*OeF3’5’H*	5’-AGTGGTCACCAATGGGATGT-3’	5’-CACATCAAACGTGGCTCATT-3’	[23]
*OeDFR*	5’-ATTTCAGGTGTTGGCTGAGG-3’	5’-ATTCCATATGGCCAGGTCAA-3’	[21]
*OeANS*	5’-GCATAGGGTCACTGTCAATGG-3’	5’-TCCTTACCATCATGGCCTTT-3’	[23]
*OeUFGT*	5’-AATGGCTTTGATGGAAGGTG-3’	5’-TTCACGCTGGCATAAACTCA-3’	[21]
*Oeu050989.1*	5′-TGGTCAAATTACTTTTCGGGAAGT-3′	5’-TCTCGTGCCATGTGTAATTATGG-3’	Our study
*Oeu004741.1*	5’-ACTGACAGACAGAACCCCATGA-3’	5’-ATCTGCAGGATTTGCATGAGAA-3’	Our study
*Oeu004739.1*	5’-TCTCTTATTTTCAACCATACACTCTTCAA-3’	5’-AGAGCATGTTTGTTTCTCATTTTCA-3’	Our study
*OeCRY2*	5′-GTCCTACAAGCTCGTCCTATG-3′	5′-CTTGTCGCAACTATGCAAGT-3′	[55]

## Data Availability

All data used in this study appear in the paper’s text, figures, and Supplementary Material.

## Supplementary Materials

The following supporting information can be downloaded at: https://www.mdpi.com/article/10.3390/ijms24108770/s1.
